# Do mobile phones and laptop computers really impact sperm?

**DOI:** 10.1080/20905998.2024.2381957

**Published:** 2024-07-21

**Authors:** Parviz K. Kavoussi, Shahryar K. Kavoussi

**Affiliations:** Department of Reproductive Urology, Austin Fertility & Reproductive Medicine/Westlake IVF, Austin, TX, USA

**Keywords:** Cell phone, computers, infertility, male, sperm

## Abstract

Over the past few decades, the male population’s fertility has decreased as demonstrated by an overall worsening of semen analysis parameters. Environmental variables are undoubtedly contributing elements. The technology that people rely on to enhance their quality of life and efficiency in many facets of daily life may also have pathologic consequences on the testicles and affect semen characteristics. Mobile phones and laptop computers are two frequently hypothesized sources of electromagnetic radiation and heat that can interfere with male fertility. These are two of the most widely utilized devices available today, and the majority of men use one or the other of them for the majority of their work.

Understanding these consequences, as well as their possible influence and severity on male reproductive capacity, is the aim of this review of the medical literature. A thorough analysis of the most recent research in medicine, including original investigations, systematic reviews, and meta-analyses, was carried out by searching PubMed for publications on the relationship between male fertility and laptops and cell phones. A definitive understanding of the impact remains elusive due to contradicting data in the medical literature, despite some studies suggesting a possible negative influence of laptop computers and cell phones on male fertility.

Based on the available data in the medical literature, it is debatable whether laptop computers and cell phones have a negative effect on a man’s fertility. Nevertheless, men who want to be cautious and rule out any potential factors that can affect fertility may decide to reduce or avoid these exposures.

## Introduction

Estimated 48.5 million couples worldwide suffer from infertility, making it an extremely common challenge [1]. Infertility affects approximately 15% of the couples in the US after they tried to conceive through unprotected intercourse for a year without success. Of these infertile couples, 20% have a male factor alone and in 40%, it is a contributing factor in addition to female factors as causes, indicating that male factor involvement is a contributory culprit in 60% of the infertile couples [2]. The semen analysis parameters have declined with time, according to numerous research studies [3–5]. While there are other theories as to why male fertility has decreased over time, including a growing population of older fathers, exposure to chemicals from industry, elevated rates of obesity, bad eating and other lifestyle habits, mass manufacturing of products, and marijuana use; environmental factors, including the usage of relevant technologies like laptops and cell phones, cannot be disregarded as potential contributory culprits [6]. The majority of individuals now use their cell phones and laptop computers on a daily basis. However, because of their possible negative effects on male fertility, the impacts of radiofrequency electromagnetic radiation (RF-EMR) and hyperthermia caused by these devices are still a topic of discussion and concern.

In the past 25 years, laptops have grown in acceptance and use among a wider range of people, including educators, researchers, businessmen, office workers, students, and people in general. Because laptops are lightweight and portable, they are easier to use, which has increased their popularity. Millions of men, including those who are fertile, use laptop computers on a regular basis for extended periods of time. Thirty-two percent of university students reported using a laptop for one to 2 hours a day, accounting for an estimated 88% of laptop computer users [7]. There are concerns that using a laptop computer can negatively affect testicular and sperm functions due to a variety of factors, such as heat and electromagnetic fields produced by the computer’s internal electronic circuitry.

As common as laptop computer use is, cell phone use or at least being on a man’s person is extremely common and is arguably trending towards being universal in developed countries. This implicates cell phones for many men as a daily, constant during waking hours, exposure to the man’s body. As the front pockets of trousers tend to be a popular location to store cell phones, they are in close proximity to the man’s reproductive organs.

The aim of this manuscript is to review the data available in animal models and human studies on whether laptop computers and cell phones have an adverse impact on sperm or not, and if a consensus may be reached for a clinical recommendation.

## Methods

A comprehensive literature review was performed using the Pubmed database. The search terms used included laptop computer, mobile phone, cellular phone, cell phone, sperm, and male fertility. All human in vitro studies, animal experimental studies, human observational studies, review articles, full systemic review and meta-analyses were included. This included a total of 41 published studies. Screening was performed by an independent reviewer.

## Results

### Laptop computers

Testicular hyperthermia is the main theory on the possible negative effect of laptop computers on spermatozoa. Published in 2005 [8], the first study to demonstrate elevated scrotal temperature as a result of laptop computer use raised concerns about the potential effects on spermatogenesis. Subsequent research in 2010 revealed comparable results, suggesting that the internal components of laptop computers produce enough heat to elevate the temperature of the scrotum [9]. Significantly elevated scrotal temperatures were seen in 29 healthy individuals throughout two separate 60-minute laptop computer sessions [10]. A follow-up study showed that using a functional laptop computer on the lap cannot be adjusted to not raise scrotal temperatures, independent of leg position or use of a lap pad. Unfortunately, it is impossible to stop the rise in scrotal heat that occurs when a laptop computer is in use on one’s lap. Using a laptop computer, 29 healthy volunteers had their scrotal temperatures taken over the course of three 60-minute sessions. First, after using a laptop for 11 minutes, while seated with legs roughly parallel and a scrotal temperature rise of 2.3°C to 2.5°C. Second, after 11 minutes of laptop use, the temperature increased by 2.1◦ C with the legs closely spaced apart and a lap pad beneath the operating laptop computer. Third, using a lap pad beneath the laptop computer while seated with legs spread at a 70° angle raised the scrotal temperatures by 1.4°C in just 28 minutes after using the laptop. Sitting upright or using a lap pad will not stop scrotal hyperthermia when using a functioning laptop computer, but these maneuvers can lessen such risk [10]. Neither correlative semen parameters nor quality parameters of sperm were tested in any of the investigations measuring scrotal temperatures.

The possible influence of wireless internet is another potential source of harm that laptop computers may cause to spermatozoa. In a prospective study, semen samples from donor sperm samples, which were mostly normozoospermic, were separated into two aliquots. One aliquot was exposed ex vivo to a laptop connected to the internet via wireless technology for 4 hours, while the other aliquot from the same sample served as a control. The results showed that the aliquot from the wireless internet-connected laptop had significantly lower sperm motility and higher rates of sperm DNA fragmentation than the control (SDF) [11]. This study’s methodology was not designed to distinguish between the negative impact on ex vivo spermatozoa resulting from wireless internet use versus a thermal effect from the laptop [12]. Electromagnetic fields have also been suggested as a potential source of harm to spermatozoa [7].

### Cell phones

While there is a dearth of information regarding the use of laptop computers and male fertility, there is a wealth of research on the possible effects of cell phones on male fertility, mostly in animal and in vitro models, with a small number of human clinical studies. Rats exposed to cell phones in the ‘speech position’ and the ‘standby’ position were found to have smaller seminiferous tubules than the sham group in a 1999 study using a murine model [13]. Additionally, the ‘speech’ group’s rectal temperature was found to be higher than that of the sham and ‘standby’ groups. The morphology or quantity of sperm did not differ. The authors assumed that the exposure to microwaves from the devices may be the cause of these effects [13].

Since then, research on rats exposed to mobile phones has shown that exposure to the device worsens traditional semen parameters and spermatogenesis by histopathology [14–16]. Cell phone use has been linked to higher levels of oxidative stress, apoptosis, and seminal reactive oxygen species in several murine model studies [14,17–19]. While some studies have found no effects of radiofrequency exposure to rat reproductive tissue, long-term exposure of rats to cell phones has been shown to impact the histopathology of the testis, increasing the rate of hypospermatogenesis and maturation arrest patterns [20,21].

Another study on rats revealed contradictory findings, indicating that sub-chronic exposure to radio frequency from cell phones had no negative effects on rat spermatogenesis [22]. There was no negative effect of mobile phone exposure on spermatozoa, semen parameters, or DNA integrity, according to other research conducted on mice [23,24]. Regardless of the degree of exposure to 3rd generation (3 G) cellular phone RF-EMR to mice, there was no difference in fertilization, embryogenesis, or blastocyst formation in one study [25]. This included a group whose gametes were exposed to a level 100 times higher than that of human spermatozoa on a daily basis.

There was no change in sperm count, but the exposed aliquot of semen had deteriorated motility metrics, according to an in vitro investigational study that compared aliquots of semen samples subjected to an activated 900 MHz cellular phone and an aliquot used as a control which was not exposed [26]. It was deduced that the cellular phone’s RF-EMR emissions were the cause of this effect. Similar results of deteriorated semen parameters after semen exposure to cell phones have been reported in other in vitro studies [27–31]. Other in vitro investigations have gone beyond the standard criteria of the conventional semen analysis parameters to demonstrate the possible negative effects of RF-EMR from cell phones on higher-level factors such as sperm DNA damage, apoptosis, reactive oxygen species, and overall antioxidant capacity [28,29,31–33].

According to one in vitro study, exposure of semen to cell phone did not negatively impact the acrosome reaction, but it did cause a notable decrease in the amount of sperm head area and the percentage of acrosomes in the head area when compared to controls. Furthermore, sperm binding to hemizona significantly decreased [34]. This study’s validity has drawn criticism [35].

There have been contradictory results from other in vitro studies. For example, there was no evidence that cell phone exposure had a negative effect on caspase 3 activity, phosphatidylserine externalization, sperm DNA strand breaks, or the production of reactive oxygen species [36].

In a clinical trial, 361 men undergoing fertility examination were divided into groups according to three distinct time frames of daily exposure to cell phones and whether they used them. Men who were exposed to cell phones for extended periods of time showed a duration-dependent decline in their sperm count, overall motility, viability, and normal morphology [37]. Other studies have shown a correlation between the duration and use of cell phones and the worsening of various semen metrics [38–41] as well as other higher-order factors including sperm DNA damage rates [42]. Conversely, no negative effect of mobile phone exposure or length of exposure has been observed on conventional semen parameters in other human studies [42,43].

According to a 2018 review, radiofrequency electromagnetic fields from laptops and cell phones affect cellular metabolism, endocrine kinases, and genomic instability, in addition to negatively affecting conventional semen parameters [44]. The mini-review, which was released in 2020 makes the case that wireless technologies, laptops, and cell phones that cause scrotal hyperthermia and oxidative stress affect spermatozoa in both humans and animals by decreasing motility and causing structural abnormalities that appear to be correlated with the duration of testicular exposure to these environmental factors [45].

## Discussion

It is widely known and supported by a large body of research that scrotal hyperthermia is associated with lower semen parameters and male infertility via oxidative stress, sperm DNA integrity damage, negative effects on semen parameters and germ cell apoptosis [46–48]. It has also been demonstrated that external environmental conditions that change testicular thermoregulation and cause heat stress promote germ cell apoptosis, which in animal models can even result in a decrease in testicular weight, as well as altered DNA synapses and strand breakage. Reduced sperm-zona pellucida binding and oocyte penetrating capacity are observed in epididymal spermatozoa under heat stress in mice [49]. Research has shown that using a laptop computer raises scrotal temperatures, however correlational studies have not examined the effect on the reproductive markers that can be measured.

Concerns about the RF-EMR released by cell phones and their possible effects on sperm have grown as a result of their increased use over the past 25 years [50]. Nearby relay base stations or antennas receive RF-EMR emissions from cellular phones. Human bodies function as antennae, taking in radiation and transforming it into alternate eddy currents. Due to the low-frequency microwave range (800–2200 MHz) of these radio waves, the energy emitted is insufficient to break the chemical bonds in the biological system, protecting human tissues from significant harm [51].

Speaking into a cell phone causes sound waves to travel via a transmitter, which then transforms the sound into a sine wave which is a signal sent to the antenna, which in turn sends the sine wave signal out in all directions into space. This electric sine wave current passing through the transmitter, the current produces an electromagnetic field around it, and as this field moves back and forth, the electromagnetic field continues to build and collapse which forms electromagnetic radiation. Technological developments in cellular phone telecommunication systems raise the frequency of the transmission and consequently the energy of radiofrequency waves [51].

This electric sine wave current passing through the transmitter, the current produces an electromagnetic field around it, and as this field moves back and forth, the electromagnetic field continues to build and collapse which forms electromagnetic radiation. There are mixed results, despite the fact that most rodent research has demonstrated a negative effect on sperm parameters and elevated oxidative stress. There have mainly been two experimental methods used to investigate the potential impact on humans. Two approaches have been used to research the effects of mobile phone exposure directly on semen: one involves studying the impacts in vitro, while the other involves comparing men who use and do not use cell phones in terms of sperm parameters. Some studies have also evaluated the effects based on the duration of exposure. There have been conflicting findings as well, although the majority of data have demonstrated deteriorated semen parameters and other aspects affecting spermatozoa with exposure and with longer periods of exposure [23,36,52].

### Challenges and limitations of research on the topic

Regarding the impact of mobile phone and laptop exposure on male fertility, there is evident disagreement in the medical literature (Table 1). A conclusive response on this subject is elusive due to the contradictory data that is currently accessible. The challenge is that it is very hard to design credible, repeatable studies on this contentious topic. In an attempt to reduce factors that could potentially negatively impact spermatozoa, we cannot ask our male patients who are trying to conceive to live in a bubble, and the data is not entirely clear, but it might be wise to suggest using desktop computers or laptops on desks rather than on one’s body, and avoiding carrying cell phones in the front pockets of trousers as reasonably simple actions that infertile patients can do to eliminate these potential variables, while the actual benefit of doing so has not been proven with enough data to support consensus recommendations.

**Table 1.** Summary of the 41 publications included in the review including the lead author as a reference, the type of device sperm were exposed to (LC = laptop computer, CP = cellular phone), the study design and whether the study reported an adverse impact or no adverse impact and the main findings. The results are color coded by adverse effects (yellow), not adverse effects (green), and equivocal findings (blue).

Study Reference	Device	Study Design	Results/Main finding
Sheynkin et al., 2012	LC	Human, observational	Adverse, increase temperature
Isele et al., 2010	LC	Human, observational	Adverse, increase temperature
Sheynkin et al., 2011	LC	Human, observational	Adverse, increase temperature
Avendado et al., 2012	LC	Human, in vitro	Adverse, decrease progressive motility, increase SDF
Dore et al., 2012	LC	Human, in vitro	Adverse, decrease motility, increase SDF
Mortazavi et al., 2016	LC	Review	Adverse, increase temperature, worsen sperm quality
Dasdag et al., 1999	CP	Animal, experimental	Equivocal
Ghanbari et al., 2013	CP	Animal, experimental	Adverse, worsen sperm parameters and total antioxidant capacity
Kesari et al., 2011	CP	Animal, experimental	Adverse, increased free radicals
Kesari et al., 2010	CP	Animal, experimental	Adverse, decreased sperm count, increase apoptosis
Shahin et al., 2018	CP	Animal, experimental	Adverse, increased oxidative stress and apoptosis
Meo et al., 2011	CP	Animal, experimental	Adverse, hypospermatogenesis and maturation arrest
Trosic et al., 2013	CP	Animal, experimental	Not adverse, no change in sperm quantity, quality, or morphology
Lee et al., 2010	CP	Animal, experimental	Not adverse, no change in epididymal sperm counts or testicular apoptosis
Imai et al., 2011	CP	Animal, experimental	Not adverse, no change in testis, epididymis, seminal vesicle, or prostate weight, no decrease in sperm counts in testis or epididymis
Vereschako et al., 2017	CP	Animal, experimental	Adverse, decrease in viability of mature germ cells
Suzuki et al., 2017	CP	Animal, experimental	Not adverse, no change in fertilization, embryogenesis, and blastocyst formation
Erogul et al., 2006	CP	Human, in vitro	Adverse, worsens motility
Falzone et al., 2008	CP	Human, in vitro	Not adverse, no change in mitochondrial membrane potential or motility
Zalata et al., 2015	CP	Human, in vitro	Adverse, decrease motility and acrosin activity, increase in SDF and seminal clusterin gene expression
Wang et al., 2015	CP	Human, in vitro	Adverse, decrease motility and viability and increase sperm head defects and early apoptosis
Zhang et al., 2016	CP	Human, observational	Adverse, decrease semen volume, sperm concentration, and sperm count
Hassanzadeh et al., 2022	CP	Human, in vitro	Adverse, decrease viability and motility, increase SDF and apoptosis
Agarwal et al., 2009	CP	Human, in vitro	Adverse, decrease motility and viability, increase ROS
De Iuliis et al., 2009	CP	Human, in vitro	Adverse, increase ROS and SDF
Falzone et al., 2011	CP	Human, in vitro	Adverse, worsened morphometrics, decrease sperm binding to hemizona
Falzone et al., 2010	CP	Human, in vitro	Not adverse, no change in caspace 3 activity, phosphatidylserine expression, SDF, or ROS
Agarwal et al., 2008	CP	Human, observational	Adverse, decrease sperm count, motility, viability, and normal morphology
Wdowiak et al., 2007	CP	Human, observational	Adverse, decrease progressive motility and normal morphology
Gutschi et al., 2011	CP	Human, observational	Adverse, decrease normal morphology
Kim et al., 2021	CP	Systemic review and meta-analysis	Adverse, decrease concentration, motility, and viability
Zhang et al., 2022	CP	Human, observational	Adverse, decrease motility
Rago et al., 2013	CP	Human, observational	Adverse, increase SDF
Hatch et al., 2021	CP	Human, observational	Not adverse, no significant change in fecundability
Kesari et al., 2018	LC/CP	Review	Adverse, increase ROS
Okechukwu et al., 2020	CP	Review	Adverse, decrease motility, increased sperm structural abnormalities and ROS
Gao et al., 2022	LC/CP	Review	Adverse, heat stress
Durairajanayagam et al., 2015	LC	Review	Adverse, heat stress
Gorpinchenko et al., 2014	CP	Human, in vitro	Adverse, decrease motility, increase SDF
Agarwal et al., 2011	CP	Review	Equivocal
La Vignera, 2012	CP	Review	Adverse, decrease sperm count, motility, and normal morphology, and viability, increase OS

*Abbreviations: LC = laptop computer, CP = cellular phone, SDF = sperm DNA fragmentation, ROS = reactive oxygen species.

## Conclusions

Based on the data currently available in the medical literature, it appears that the effects of laptops and cell phones on a man’s fertility are still up for debate. Nevertheless, men who want to be safe and rule out any potential factors that might affect their fertility may decide to reduce these exposures. Well-designed research is still required to help reach a more conclusive knowledge and enable more precise patient counseling.

## References

[1] Mascarenhas MN, Flaxman SR, Boerma T, et al. National, regional, and global trends in infertility prevalence since 1990: a systematic analysis of 277 health surveys. PLoS Med. 2012;9(12):e1001356. doi: 10.1371/journal.pmed.1001356 23271957 PMC3525527

[2] Thonneau P, Marchand S, Tallec A, et al. Incidence and main causes of infertility in a resident population (1 850 000) of three French regions (1988–1989). Hum Reprod. 1991;6(6):811–816. doi: 10.1093/oxfordjournals.humrep.a137433 1757519

[3] Tiegs AW, Landis J, Garrido N, et al. Total motile sperm count trend over time: evaluation of semen analyses from 119,972 men from subfertile couples. Urology. 2019;132:109–116. doi: 10.1016/j.urology.2019.06.038 31326545

[4] Sengupta P, Borges E, Dutta S, et al. Decline in sperm count in European men during the past 50 years. Hum Exp Toxicol. 2018;37(3):247–255. doi: 10.1177/0960327117703690 28413887

[5] Rolland M, Le Moal J, Wagner V, et al. Decline in semen concentration and morphology in a sample of 26 609 men close to general population between 1989 and 2005 in France. Hum Reprod. 2013;28(2):462–470. doi: 10.1093/humrep/des415 23213178 PMC4042534

[6] Mann U, Shiff B, Patel P. Reasons for worldwide decline in male fertility. Curr Opin Urol. 2020;30(3):296–301. doi: 10.1097/MOU.0000000000000745 32168194

[7] Mortazavi SA, Taeb S, Mortazavi SMJ, et al. The fundamental reasons why laptop computers should not be used on your lap. J Biomed Phys Eng. 2016;6(4):279–284. doi: 10.1016/j.beem.2010.08.006 28144597 PMC5219578

[8] Sheynkin Y, Jung M, Yoo P, et al. Increase in scrotal temperature in laptop computer users. Hum Reprod. 2005;20(2):452–455. doi: 10.1093/humrep/deh616 15591087

[9] Isele H. Eierwärmer. MMW - Fortschr der Medizin. 2010;152(51–52):17. doi: 10.1007/BF03367560 21298817

[10] Sheynkin Y, Welliver R, Winer A, et al. Protection from scrotal hyperthermia in laptop computer users. Fertil Steril. 2011;95(2):647–651. doi: 10.1016/j.fertnstert.2010.10.013 21055743

[11] Avendano C, Mata A, Sanchez Sarmiento CA, et al. Use of laptop computers connected to internet through wi-fi decreases human sperm motility and increases sperm DNA fragmentation. Fertil Steril. 2012;97(1):39–45.e2. doi: 10.1016/j.fertnstert.2011.10.012 22112647

[12] Dore JF, Chignol MC. Laptop computers with wi-fi decrease human sperm motility and increase sperm DNA fragmentation. Fertil Steril. 2012;97(4):e12. doi: 10.1016/j.fertnstert.2012.01.102 22306709

[13] Dasdag S, Ketani MA, Akdag Z, et al. Whole-body microwave exposure emitted by cellular phones and testicular function of rats. Urol Res. 1999;27(3):219–223. doi: 10.1007/s002400050113 10422825

[14] Ghanbari M, Mortazavi SB, Khavanin A, et al. Simultaneous effects of exposure to microwaves and noise on male rats’ sperm parameters and total antioxidant capacity. Int J Fertil Steril. 2013;1(4):21–28. doi: 10.5812/jhs.8230 PMC3850324 24520459

[15] Oh JJ, Byun S-S, Lee SE, et al. Effect of electromagnetic waves from mobile phones on spermatogenesis in the era of 4G-LTE. Biomed Res Int. 2018;2018:1–8. doi: 10.1155/2018/1801798 PMC5896334 29789776

[16] Gautam R, Singh KV, Nirala J, et al. Oxidative stress-mediated alterations on sperm parameters in male Wistar rats exposed to 3G mobile phone radiation. Andrologia. 2019;51(3):e13201. doi: 10.1111/and.13201 30461041

[17] Kesari KK, Kumar S, Behari J. Effects of radiofrequency electromagnetic wave exposure from cellular phones on the reproductive pattern in male Wistar rats. Appl Biochem Biotechnol. 2011;164(4):546–559. doi: 10.1007/s12010-010-9156-0 21240569

[18] Kesari KK, Kumar S, Behari J. Mobile phone usage and male infertility in Wistar rats. Indian J Exp Biol. 2010;48(10):987–992. 21299041

[19] Shahin S, Singh SP, Chaturvedi CM. 1800 MHz mobile phone irradiation induced oxidative and nitrosative stress leads to p53 dependent Bax mediated testicular apoptosis in mice, Mus musculus. J Cell Physiol. 2018;233(9):7253–7267. doi: 10.1002/jcp.26558 29637556 PMC13482464

[20] Meo SA, Arif M, Rashied S, et al. Hypospermatogenesis and spermatozoa maturation arrest in rats induced by mobile phone radiation. J Coll Physicians Surg Pak. 2011;21(5):262–265. 21575531

[21] Trosic I, Mataušić-Pišl M, Pavičić I, et al. Histological and cytological examination of rat reproductive tissue after short-time intermittent radiofrequency exposure. Arh Hig Rada Toksikol. 2013;64(4):513–519. doi: 10.2478/10004-1254-64-2013-2394 24384757

[22] Lee HJ, Pack J-K, Kim T-H, et al. The lack of histological changes of CDMA cellular phone-based radio frequency on rat testis. Bioelectromag. 2010;31(7):528–534. doi: 10.1002/bem.20589 20607737

[23] Imai N, Kawabe M, Hikage T, et al. Effects on rat testis of 1.95-GHz W-CDMA for IMT-2000 cellular phones. Syst Biol Reprod Med. 2011;57(4):204–209. doi: 10.3109/19396368.2010.544839 21204746

[24] Vereschako GG, Chueshova NV. Reaction of reproductive system and epididymal spermatozoa Of rats to electromagnetic radiation from mobile phone (1745 MHz) of various duration. Radiats Biol Radioecol. 2017;57(1):71–76. 30698934

[25] Suzuki S, Okutsu M, Suganuma R, et al. Influence of radiofrequency–electromagnetic waves from 3rd-generation cellular phones on fertilization and embryo development in mice. Bioelectromag. 2017;38(6):466–473. doi: 10.1002/bem.22063 28628221

[26] Erogul O, Oztas E, Yildirim I, et al. Effects of electromagnetic radiation from a cellular phone on human sperm motility: an in vitro study. Arch Med Res. 2006;37(7):840–843. doi: 10.1016/j.arcmed.2006.05.003 16971222

[27] Falzone N, Huyser C, Fourie F, et al. In vitro effect of pulsed 900 MHz GSM radiation on mitochondrial membrane potential and motility of human spermatozoa. Bioelectromag. 2008;29(4):268–276. doi: 10.1002/bem.20390 18163440

[28] Zalata A, El-Samanoudy AZ, Shaalan D, et al. In vitro effect of cell phone radiation on motility, DNA fragmentation and clusterin gene expression in human sperm. Int J Fertil Steril. 2015;9(1):129–136. doi: 10.22074/ijfs.2015.4217 25918601 PMC4410031

[29] Wang D, Li B, Liu Y, et al. Impact of mobile phone radiation on the quality and DNA methylation of human sperm in vitro. Zhonghua Nan Ke Xue. 2015;21(6):515–520. 26242041

[30] Zhang G, Yan H, Chen Q, et al. Effects of cell phone use on semen parameters: results from the MARHCS cohort study in Chongqing, China. Environ Int. 2016;91:116–121. doi: 10.1016/j.envint.2016.02.028 26949865

[31] Hassanzadeh-Taheri M, Khalili MA, Hosseininejad Mohebati A, et al. The detrimental effect of cell phone radiation on sperm biological characteristics in normozoospermic. Andrologia. 2022;54(1):e14257. doi: 10.1111/and.14257 34628682

[32] Agarwal A, Desai NR, Makker K, et al. Effects of radiofrequency electromagnetic waves (RF-EMW) from cellular phones on human ejaculated semen: an in vitro pilot study. Fertil Steril. 2009;92(4):1318–1325. doi: 10.1016/j.fertnstert.2008.08.022 18804757

[33] De Iuliis GN, Newey RJ, King BV, et al. Mobile phone radiation induces reactive oxygen species production and DNA damage in human spermatozoa in vitro. PLOS ONE. 2009;4(7):e6446. doi: 10.1371/journal.pone.0006446 19649291 PMC2714176

[34] Falzone N, Huyser C, Becker P, et al. The effect of pulsed 900-MHz GSM mobile phone radiation on the acrosome reaction, head morphometry and zona binding of human spermatozoa. Int J Androl. 2011;34(1):20–26. doi: 10.1111/j.1365-2605.2010.01054.x 20236367

[35] Lerchl A. Letter on ‘the effect of pulsed 900-MHz GSM mobile phone radiation on the acrosome reaction, head morphometry and zona binding of human spermatozoa’ by Falzone et al. (Int J Androl 34: 20–26, 2011). Int J Of Androl. 2012;35(1):103–103. doi: 10.1111/j.1365-2605.2011.01182.x 20236367

[36] Falzone N, Huyser C, Franken DR, et al. Mobile phone radiation does not induce pro-apoptosis effects in human spermatozoa. Radiat Res. 2010;174(2):169–176. doi: 10.1667/RR2091.1 20681783

[37] Agarwal A, Deepinder F, Sharma RK, et al. Effect of cell phone usage on semen analysis in men attending infertility clinic: an observational study. Fertil Steril. 2008;89(1):124–128. doi: 10.1016/j.fertnstert.2007.01.166 17482179

[38] Wdowiak A, Wdowiak L, Wiktor H. Evaluation of the effect of using mobile phones on male fertility. Ann Agric Environ Med. 2007;14(1):169–172. 17655195

[39] Gutschi T, Mohamad Al-Ali B, Shamloul R, et al. Impact of cell phone use on men’s semen parameters. Andrologia. 2011;43(5):312–316. doi: 10.1111/j.1439-0272.2011.01075.x 21951197

[40] Kim S, Han D, Ryu J, et al. Effects of mobile phone usage on sperm quality – no time-dependent relationship on usage: a systematic review and updated meta-analysis. Environ Res. 2021;202:111784. doi: 10.1016/j.envres.2021.111784 34333014

[41] Zhang S, Mo F, Chang Y, et al. Effects of mobile phone use on semen parameters: a cross-sectional study of 1634 men in China. Reprod Fertil Dev. 2022;34(9):669–678. doi: 10.1071/RD21234 35436442

[42] Rago R, Salacone P, Caponecchia L, et al. The semen quality of the mobile phone users. J Endocrinol Invest. 2013;36(11):970–974. doi: 10.3275/8996 23722985

[43] Hatch EE, Willis SK, Wesselink AK, et al. Male cellular telephone exposure, fecundability, and semen quality: results from two preconception cohort studies. Hum Reprod. 2021;36(5):1395–1404. doi: 10.1093/humrep/deab001 33564831 PMC8058594

[44] Kesari KK, Agarwal A, Henkel R. Radiations and male fertility. Reprod Biol Endocrinol. 2018;16(1):118. doi: 10.1186/s12958-018-0431-1 30445985 PMC6240172

[45] Okechukwu CE. Does the use of mobile phone affect male fertility? A mini-review. J Hum Reprod Sci. 2020;13(3):174–183. doi: 10.4103/jhrs.JHRS_126_19 33311902 PMC7727890

[46] Gao Y. Covid continues, science advances, a new year of SBiRM begins. Syst Biol Reprod Med. 2022;68(1):1–2. doi: 10.1080/19396368.2022.2017733 35125042

[47] Rao M, Xia W, Yang J, et al. Transient scrotal hyperthermia affects human sperm DNA integrity, sperm apoptosis, and sperm protein expression. Androl. 2016;4(6):1054–1063. doi: 10.1111/andr.12228 27410176

[48] Rao M, Zhao X-L, Yang J, et al. Effect of transient scrotal hyperthermia on sperm parameters, seminal plasma biochemical markers, and oxidative stress in men. Asian J Androl. 2015;17(4):668–675. doi: 10.4103/1008-682X.146967 25652627 PMC4492061

[49] Durairajanayagam D, Agarwal A, Ong C. Causes, effects and molecular mechanisms of testicular heat stress. Reprod Biomed Online. 2015;30(1):14–27. doi: 10.1016/j.rbmo.2014.09.018 25456164

[50] Gorpinchenko I. Commenting on Gorpinchenko et al the influence of direct mobile phone radiation on sperm quality. Cent European J Urol. 2014;67(4):65–71. doi: 10.5173/ceju.2014.04.art26 PMC4074720 24982785

[51] Agarwal A, Singh A, Hamada A, et al. Cell phones and male infertility: a review of recent innovations in technology and consequences. Int Braz J Urol. 2011;37(4):432–454. doi: 10.1590/S1677-55382011000400002 21888695

[52] La Vignera S, Condorelli RA, Vicari E, et al. Effects of the exposure to mobile phones on male reproduction: a review of the literature. J Androl. 2012;33(3):350–356. doi: 10.2164/jandrol.111.014373 21799142

